# Parametrization of Linear Vibronic Coupling Models for Degenerate Electronic States

**DOI:** 10.1021/acs.jpca.4c07472

**Published:** 2025-03-04

**Authors:** Dilara Farkhutdinova, Severin Polonius, Paul Karrer, Sebastian Mai, Leticia González

**Affiliations:** † Institute of Theoretical Chemistry, Faculty of Chemistry, University of Vienna, Währinger Straße 17, 1090 Vienna, Austria; ‡ Vienna Doctoral School in Chemistry (DoSChem), University of Vienna, Währinger Straße 42, 1090 Vienna, Austria; ¶ Research Platform on Accelerating Photoreaction Discovery (ViRAPID), University of Vienna, Währinger Strasse 17, 1090 Vienna, Austria

## Abstract

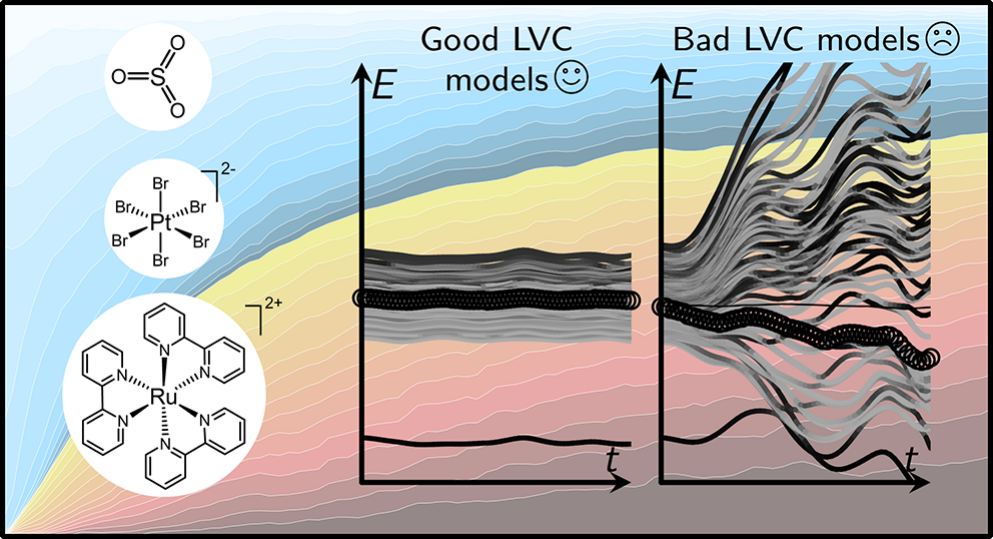

Linear vibronic coupling (LVC) models have proven to be effective in describing coupled excited-state potential energy surfaces of rigid molecules. However, obtaining the LVC parameters in molecules with many degrees of freedom and a large number of, possibly (near-)degenerate, electronic states can be challenging. In this paper, we discuss how the linear intra- and interstate couplings can be computed correctly using a numerical differentiation scheme, requiring a phase correction and sufficient numerical precision in the involved electronic structure calculations. The numerical scheme is applied to three test systems with symmetry-induced state degeneracies: SO_3_, [PtBr_6_]^2–^, and [Ru(bpy)_3_]^2+^. The first two systems are employed to validate the performance of the parametrization scheme. LVC potentials for SO_3_ are shown to reproduce the trigonal symmetry of the potential energy surfaces. The integration of the LVC potentials for [PtBr_6_]^2–^ with the surface-hopping trajectory method illustrates how spurious parameters lead to erroneous trajectory behavior. In the transition metal complex [Ru(bpy)_3_]^2+^, extensive nonadiabatic simulations using LVC potentials are compared to those conducted with direct on-the-fly potentials. The simulations with LVC potentials demonstrate excellent agreement with the on-the-fly results while incurring costs that are 5 orders of magnitude lower. Further, the simulations evidence that intersystem crossing in [Ru(bpy)_3_]^2+^ occurs at a slightly slower rate than luminescence decay, underscoring the importance of simulating the actual experimental observable when comparing computed time constants with experimental time constants. Lastly, the initial nuclear response to excitation involves a rapid, short-lived, and small elongation of the Ru–N bonds, with no charge localization occurring on a sub-ps time scale.

## Introduction

Modeling nonadiabatic dynamics is essential for understanding molecular behavior in light-induced reactions, fundamental to applications such as solar energy conversion, catalysis, and molecular electronics.^1−3^ Since light irradiation triggers processes where the nuclear motion is influenced by two or more electronic states, it is important to capture the interactions between electronic and nuclear motions properly.^4,5^ Thus, performing nonadiabatic dynamics simulations requires accurate potential energy surfaces (PESs) and electron–nuclear couplings. Due to the curse of dimensionality, *a priori* computing high-dimensional PESs for large molecules is unfeasible; therefore, many simulations employ “on-the-fly” methods, which evaluate the PESs during the propagation. A widely used on-the-fly method is trajectory surface hopping (SH),^6−8^ which is, e.g., implemented in our SHARC (surface hopping including arbitrary couplings) package.^9,10^

On the downside, on-the-fly nonadiabatic dynamics simulations (like SH) are limited by the computational effort required to solve the electronic Schrödinger equation for multiple states at every time step. Much effort has been made in the last decades to circumvent this problem, e.g., using machine learning^11,12^ or interpolation techniques.^13,14^ A cost-efficient alternative is to describe the PESs with vibronic coupling models.^15^ For rigid molecules (i.e., without strong anharmonicities, dissociations, floppy torsional modes, or other large-amplitude modes), a linear vibronic coupling (LVC) model can efficiently represent the coupled PESs of several excited states. We pioneered the implementation of LVC models for SH within our SHARC package^16^ and have illustrated different parametrizations of LVC models from electronic structure calculations.^16,17^ The combination of SH and LVC has proven highly efficient for describing nonadiabatic dynamics in rigid organic molecules and transition metal complexes involving multiple states,^18^ with speed-ups of several orders of magnitude relative to on-the-fly electronic structure calculations. This enormous speed-up has enabled extending the propagation times,^19^ including thousands of trajectories,^20^ using expensive multiconfigurational PESs,^21^ and testing the influence of different parameters on the dynamics^22,23^ very efficiently, even in the presence of laser fields.^24^

The semiautomatic parametrization of LVC models within the SHARC package requires, besides energies and gradients, either the nonadiabatic coupling vectors^16^ or the computation of wave function overlaps between the reference geometry and displaced geometries.^17^ The numerical differentiation scheme using wave function overlaps has been used very successful for systems ranging from SO_2_ to large transition metal complexes.^18^ However, in a previous publication,^25^ some of us noted that erroneous coupling parameters can be obtained if states are degenerate at the reference geometry due to symmetry. Hence, in the present contribution, we have reassessed the numerical parametrization scheme for degenerate states, incorporating a more accurate phase correction algorithm.^26^ Additionally, we have evaluated the impact of the precision of the electronic structure calculations involved and established the importance of selecting accurate displacement sizes during the parametrization process.

Using the new phase correction and following the precision recommendations, LVC models for three test systems were investigated: SO_3_, [PtBr_6_]^2–^, and [Ru(bpy)_3_]^2+^. The SO_3_ molecule has been chosen as a small example molecule with *D*_3*h*_ symmetry, for which the symmetry-imposed properties of the LVC parameters are scrutinized in detail. The [PtBr_6_]^2–^ complex has octahedral *O*_*h*_ symmetry and in our previous work,^25^ degenerate states needed to be manually reordered to obtain sensible parameters. Here, we show how correct parameters can be obtained automatically for [PtBr_6_]^2–^. As a third example, we revisit the relaxation dynamics of the *D*_3_-symmetric complex [Ru(bpy)_3_]^2+^, comparing the dynamics obtained with LVC with reference trajectories computed directly with on-the-fly time-dependent density functional theory (TDDFT).^27^

## Theory

Here, we briefly review the core aspects of LVC models and explain how parameters are obtained for degenerate reference states.

### LVC Parametrization for Degenerate Reference States

The LVC Hamiltonian matrix in the diabatic basis^15,18^ is
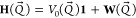
1where *Q⃗* is a vector defining the current geometry in mass-frequency-scaled normal-mode coordinates, where the coordinate of mode *i* is computed as  based on frequency ω_*i*_, transformation matrix **K**, masses *M*_*A*_ of atom *A*, and a reference geometry *r⃗*^ref^. Note that the reference geometry corresponds to *Q⃗* = 0. The term  is the reference harmonic oscillator (ω_*i*_ is the frequency of mode *i*). The elements of the vibronic coupling interaction matrix **W** are given by
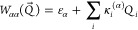
2and
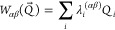
3Here, the parameter ε_α_ is the vertical energy shift of state α at *Q⃗* = 0, κ_*i*_^(α)^ gives the gradient of diabatic state α along normal mode *i* at *Q⃗* = 0, and λ_*i*_^(αβ)^ is the linear vibronic coupling between states α and β along normal mode *i*. As can be seen from eq 3, at *Q⃗* = 0 all off-diagonal elements *W*_αβ_ of **H** are zero, so the (adiabatic) eigenstates are identical to the diabatic states used to construct the LVC model.

In SHARC,^9,10^ LVC models are typically parametrized using one of two approaches. One is the “one-shot” approach,^16^ where all parameters are attained from energies, gradients, and nonadiabatic coupling vectors computed at *Q⃗* = 0. The alternative is a numerical differentiation scheme of diabatized energies that uses wave function overlaps.^17^ The latter is the standard approach when nonadiabatic coupling vectors are not available, e.g., for TDDFT or ADC(2). In this approach, for each normal mode *i*, two single-point calculations at *Q⃗* = (0, ..., ± Δ*Q*_*i*_, ...) (displacing only in mode *i*) are performed. The obtained energies (in the diagonal matrices **H**_+*i*_ and **H**_–*i*_) and the wave function overlaps to the reference geometry (**S**_+*i*_ and **S**_–*i*_) are then used to compute the matrix **λ**_*i*_ of all λ parameters of mode *i* as,
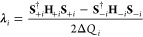
4The κ_*i*_ parameters for all states can be obtained from the diagonal of eq 4, but often are directly computed by transforming analytical gradients into the normal mode basis. We note that the outlined numerical approach^17^ is in principle based on the diabatization-by-*block*-diagonalization ansatz first proposed in 1988 by Pacher, Cederbaum, and Köppel,^28^ and later cast into various practical forms by several other groups,^29−32^ although it employs more modern wave function overlap techniques to obtain the adiabatic-to-diabatic transformation matrix **S**.

In eq 4, it is important that the overlap matrices **S**_+_ and **S**_–_ have a consistent phase convention to obtain correct parameters. Otherwise, the sign of the off-diagonal elements of **S**_+*i*_^†^**H**_+*i*_**S**_+*i*_ and **S**_–*i*_^†^**H**_–*i*_**S**_–*i*_ would become inconsistent, leading to spuriously large parameters. In previous implementations of the parametrization workflow, we employed a simple phase-correction algorithm, where only the diagonal elements *S*_*ii*_ of the overlap matrix **S** were considered. If *S*_*ii*_ < 0 and |*S*_*ii*_| > 0.5, then the column *i* of the overlap matrix is multiplied by −1. This algorithm works correctly for diagonally dominant overlap matrices, which are typically found in parametrizations done with SHARC due to the small displacements Δ*Q*_*i*_ used. However, if the states at the reference geometry are freely mixing because they are degenerate, the overlap matrices cease to be diagonally dominant and the phase correction algorithm fails, which in turn leads to erroneous parameters. For example, the overlap matrix  could be obtained if two wave functions swap their energetic order but keep their signs. This matrix is not a proper rotation matrix because its determinant is not +1, and it should be corrected to . The old phase correction algorithm would leave this matrix unchanged, as only the diagonals are taken into account (see discussion in ref (26) for more details).

As part of the present work, we have incorporated the (real-valued) phase correction algorithm of Zhou et al.^26^ into the parametrization routines of SHARC. These authors show that the overlap matrices phases should obey *parallel transport* (i.e., behave like rotation matrices), which can be achieved by swapping the phases of the columns of **S** to minimize the norm of the matrix logarithm of **S**. The algorithm automatically fixes the determinant of the matrix to +1 and leads to consistent phase conventions between all the individual displacement calculations. As we will show below, using this phase correction we do obtain correct LVC parameters also for situations where the overlap matrices are not diagonally dominant.

## Computational Details

In this section, we provide the computational details for the three test systems, SO_3_, [PtBr_6_]^2–^, and [Ru(bpy)_3_]^2+^. These include the electronic structure level of theory and settings for the LVC parametrization, the details of the nonadiabatic dynamics simulations for [PtBr_6_]^2–^ and [Ru(bpy)_3_]^2+^, and the analysis tools.

### Electronic Structure

For SO_3_ and [Ru(bpy)_3_]^2+^, the singlet ground state was computed with Kohn–Sham density functional theory using the PBE functional^33^ and the D3 dispersion correction.^34,35^ Scalar relativistic effects were included using the ZORA Hamiltonian.^36^ The ZORA-def2-SVP basis set^37^ was used throughout, except for Ru, where we employed SARC-ZORA-TZVP.^38^ To speed up the computations, the split-RIJ algorithm with the SARC/J auxiliary basis set^38^ was used. The calculations were performed in gas phase. The Tamm-Dancoff approximation (TDA) was used to obtain the electronic excited states. This setup closely mimics the approach used in our previous study of [Ru(bpy)_3_]^2+^ (PBE, D3 correction, ZORA, DZP and TZP(Ru) basis sets, TDA),^27^ because it was found to provide accurate energy gaps among the excited states, as compared to MS-CASPT2, while reproducing reasonably well the experimental absorption energies.^39^ The optimization and frequency calculation of SO_3_ was done with explicit *D*_3*h*_ symmetry in ORCA 6.0.0, while excited-state calculations of SO_3_ and all calculations of [Ru(bpy)_3_]^2+^ were done without symmetry in ORCA 5.0.4.^40^

For [PtBr_6_]^2–^, we employed the level of theory of ref (25), that is, the B3LYP functional,^41^ ZORA, the ZORA-def2-SVP^37^ and SARC-ZORA-SVP^38^ basis sets on Br and Pt, respectively, the D3 dispersion correction,^34,35^ and CPCM^42^ implicit solvation for water. The RIJCOSX approximation^43^ with the SARC/J auxiliary basis, the defgrid3 integration grid, and ”verytightscf” settings were also used. The optimization and frequency calculation was done with explicit *O*_*h*_ symmetry in ORCA 6.0.0, while remaining calculations were done without symmetry in ORCA 5.0.4.^40^

### LVC Parametrization

In SO_3_, the LVC parameters were generated for five singlet states (S_0_ plus four excited singlets) and for all six normal modes, using the level of theory described above. The numerical differentiation scheme used displacements Δ*Q* of 0.05 (in units of mass-frequency-scaled normal mode coordinates^16^). Wave function overlaps were computed from wave functions including all determinants, without any truncation. The obtained model is labeled “SO_3_ LVC”.

For [PtBr_6_]^2–^, we considered the 5*d*^6^ singlet ground state, 24 excited singlets, 24 excited triplets, and all 15 normal modes. These correspond to all possible ligand-to-metal charge transfer transitions from the 12 Br *p* orbitals (perpendicular to the Br–Pt bond) to the two Pt *e*_*g*_ orbitals. We used displacements Δ*Q* of 0.15 for modes 1–9 (68–102 cm^–1^) and 0.10 for modes 10–15 (175–215 cm^–1^). The rationale for these choices is provided below. Wave function overlaps were computed from wave functions truncated to a norm of 0.999999 (model “PtBr good”). For illustration, we also produced a second LVC model that used the same parameters, but displacements Δ*Q* of 0.05 for all modes and wave functions truncated to 0.998 (model “PtBr poor”).

For [Ru(bpy)_3_]^2+^, 177 normal modes, the singlet ground state, 17 excited singlets, and 21 excited triplets were considered. For normal modes 1–16, we used displacements of 0.15, for normal modes 17–54 displacements of 0.10, and for the remaining normal modes (55–177) the default displacements of 0.05. Wave function overlaps employed wave functions truncated to a norm of 0.9997 (model “Rubpy LVC”).

In all three systems, the ε parameters (eq 2) and the spin–orbit couplings were taken from the single-point calculations performed at the reference geometry *Q⃗* = 0. The κ parameters (eq 2) were computed from analytical gradients at the same geometry. The λ parameters (eq 3) were obtained through the numerical differentiation scheme discussed above.^17^ We note that the κ and λ parameters are separately obtained for the *M*_*S*_ = *S* component of the singlets and triplets, and subsequently used for all *M*_*S*_ values, avoiding any issues with spin degeneracy. The files containing the LVC parameters of all four models (“SO_3_ LVC” “PtBr good”, “PtBr poor”, “Rubpy LVC”) in SHARC’s file formats can be found in the Supporting Information.

### Nonadiabatic Dynamics

Nonadiabatic simulations were performed exclusively for the two transition metal complexes. For SO_3_, we focused on inspecting the symmetry of the parameters and optimizing the adiabatic *S*_4_ state, to verify that the “SO_3_ LVC” model correctly produces three symmetry-equivalent minima.

For [PtBr_6_]^2–^, we simulated a single trajectory with the goal to illustrate how inaccurate LVC parameters can lead to spurious trajectory behavior. This trajectory was started from a single initial condition sampled from the ground state Wigner distribution and excited to the adiabatic *S*_1_ state. The same trajectory was run twice, once with the “PtBr poor” and once with the “PtBr good” model. The trajectory was propagated with pySHARC^16^ for 300 fs on the diagonal potential energy surfaces,^9^ using nuclear time steps of 0.5 fs, electronic time steps of 0.02 fs, the local diabatization propagator,^44^ and an energy-based decoherence correction (α = 0.1 au).^45^ During a surface hop, the kinetic energy was adjusted by rescaling the velocity vectors.^22^

For [Ru(bpy)_3_]^2+^, we simulated two swarms of trajectories, one set using the “Rubpy LVC” model and the second with TD-PBE on-the-fly. We used the level of theory details given above, except that less strict convergence criteria (tightSCF), the defgrid2 integration grid, and a wave function truncation threshold of 0.998 were employed; as the on-the-fly trajectories do not rely on numerical differentiation, these less strict settings are adequate and make the trajectories feasible. A total of 500 initial conditions (geometries and velocities), sampled from a Wigner distribution defined by a ground state frequency calculation, was used for both swarms. At each geometry, a single point calculation was performed, and the obtained energies and oscillator strengths were used to simulate the absorption spectrum^46^ and to select the initial electronic states^47^ in the energy window of 2.5–3.0 eV. For the TDDFT simulations, 433 excited initial conditions were obtained, corresponding to 1, 9, 26, 11, 12, 11, 12, 19% starting in the adiabatic *S*_7_ to *S*_14_ states, respectively. Of these, 53 trajectories were propagated, with statistically consistent initial states (2, 6, 28, 13, 8, 9, 13, 21% in *S*_7_ to *S*_14_). For the LVC model, 582 initial conditions were obtained and all of them were used to propagate. The distribution of initial states is similar (3, 18, 34, 12, 10, 7, 6, 11% in *S*_7_ to *S*_14_ in *S*_7_ to *S*_14_), with small differences due to a small shift between the LVC and TDDFT spectra (see below). The dynamics included explicitly the ground state, 14 excited singlets, and 15 triplets. The trajectories were propagated with pySHARC^16^ for 200 fs (TDDFT) or 1000 fs (LVC) on the diagonal potential energy surfaces,^9^ using the same time steps, propagator, decoherence correction, and kinetic energy adjustment as in [PtBr_6_]^2–^. All trajectories in both swarms reached the maximum simulation time and were included in the analysis.

### Analysis

The [Ru(bpy)_3_]^2+^ trajectories were analyzed by means of the electronic populations in the molecular Coulomb Hamiltonian (MCH) representation^9^ (i.e., spin-free states). We report the quantum amplitudes transformed into this representation according to
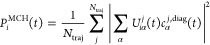
5where *c*_α_^*j*,diag^(*t*) is the wave function coefficient of state α in the diagonal basis at time *t* of trajectory *j*, *U*_*iα*_^*j*^(*t*) is an element of the diagonal–MCH transformation matrix at time *t* for trajectory *j*, and *P*_*i*_^MCH^(*t*) is the swarm-averaged population of MCH state *i* at time *t*. Additionally, we show the simulated luminescence decay, computed as the average of the oscillator strengths between active and ground state (in the diagonal representation^9^):
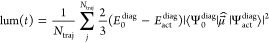
6Finally, we report the time-resolved evolution of the Ru–N, C–C (bridge between pyridines), and C–N bond lengths. To ease visualization, these were convoluted with Gaussians with full-width-at-half-maximum of 0.01 Å to obtain time-dependent density plots.

## Results and Discussion

### LVC Parameters for SO_3_

Starting with the foundational work of Jahn and Teller,^48^ there exists an extensive body of literature that deals with vibronic coupling models for systems with trigonal (or higher) symmetry, including contributions from Zgierski et al.,^49^ Bersuker,^50,51^ Köppel and Cederbaum and co-workers,^52,53^ and Yarkony.^54^ Hence, the expected form of the matrix of LVC parameters for trigonal systems is well understood, which allow us to verify whether the automatic parametrization procedure in SHARC generates parameters that correctly reproduce the symmetry of the potential energy surfaces.

SO_3_ in *D*_3*h*_ symmetry has six normal modes, which can be decomposed into the irreducible representations *a*_1_^′^ ⊕ *a*_2_^″^ ⊕ 2*e*′. The ground state S_0_ and the four lowest-lying excited states belong to the *A*_1_^′^ (*S*_0_), *A*_1_^″^ (*S*_1_), *A*_2_^′^ (*S*_2_), and *E*″ (*S*_3,4_) irreducible representations. Our focus is on the degenerate *E*″ pair. Motion along the *a* normal modes does not lift the degeneracy of the *E*″ states, and thus we only focus on the two sets of *e*′ modes. As these modes are in-plane vibrations, they can only couple the *E*″ with each other and with the *A*_1_^″^. Thus, concerning the LVC parameters that are relevant to lifting the state degeneracy, we are left with two independent (*E*″ + *A*_1_^″^) ⊗ *e*′ problems (because there are two sets of *e*′ modes).

The classical Jahn–Teller (JT) *E* ⊗ *e* problem^49−51^ can be parametrized in the following way:
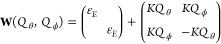
7where θ and ϕ index the two components of the *e* pair. It has been noted^50^ that the PES produced by the *E* ⊗ *e* problem produces a ‘Mexican hat‘ potential, i.e., a circle of geometries that all have the same energy, rather than a set of symmetry-equivalent local minima. Such symmetry-equivalent local minima typically arise from two types of couplings. The first type are certain quadratic vibronic coupling terms leading to the quadratic JT effect.^50^ The automatic parametrizating of such terms is rather involved^17^ and expensive, and is not the focus of the present work. Alternatively, symmetry-equivalent local minima arise by considering the pseudo-Jahn–Teller (PJT) (*E* + *A*) ⊗ *e* problem, see, e.g., ref (52):
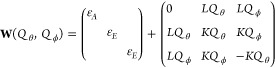
8With three arbitrary states and two arbitrary modes, we would in principle expect six independent κ and six independent λ parameters. However, due to the symmetry in the (*E* + *A*) ⊗ *e* problem, seven of these parameters are zero and the remaining parameters are all required to have magnitude *K* or *L*.

In general, normal modes and electronic states computed with quantum chemistry software will not produce degenerate *e* modes or degenerate states *E* that are properly aligned with the symmetry operations. Instead, these modes or states will be randomly rotated, which will produce an (*E* + *A*) ⊗ *e* problem with a more general shape. This can be found by applying a rotation matrix with angle ζ to the two *E* states
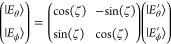
9and another rotation matrix with angle ξ to the two *e* modes
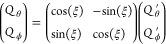
10In this case, one finds nonzero parameters
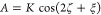
11
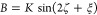
12
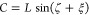
13
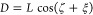
14The interaction matrix then has the following shape:
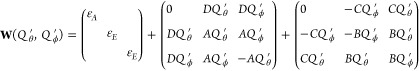
15Hence, instead of two numerically distinct parameters *K* and *L*, the LVC model will have four numerically distinct parameters *A*, *B*, *C*, and *D*, with *A*^2^ + *B*^2^ = *K*^2^ and *C*^2^ + *D*^2^ = *L*^2^. The symmetry compliance of an obtained *E* ⊗ *e* LVC model can thus be checked by asserting that |κ_*e*_1__^*E*_1_^| = |κ_*e*_1__^*E*_2_^| = |λ_*e*_2__^*E*_1_,*E*_2_^|, which needs to hold independent of how the degenerate *e* modes or degenerate states *E* are mixed. We note that, apart from a larger number of nonzero parameters, LVC models based on randomly rotated modes and states are principally equivalent to LVC models based on symmetry-aligned modes and states.

For SO_3_, at the TD-PBE level of theory given above, the obtained parameters are as follows. For the two pairs of *e*′ modes (*q*_θ_, *q*_ϕ_ at about 470 cm^–1^, *Q*_θ_, *Q*_ϕ_ at about 1300 cm^–1^), we find the following (*E*″ + *A*_1_^″^) ⊗ 2*e*′ LVC Hamiltonian parameters:
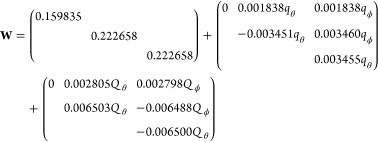
16in atomic units and only showing the upper triangle. The parameters follow the general relations for (*E* + *A*) ⊗ *e* problems to within <0.3%.

We also performed optimizations of the SO_3_ molecule to find the minima of the S_4_ adiabatic potential energy surface. This surface is the lower branch of the *E*″ state pair when moving away from the *D*_3*h*_ geometry. Using the full “SO_3_ LVC” model, we obtained three equivalent, degenerate minima that are of *C*_2*v*_ symmetry, with one long and two short bonds, and one wide and two acute angles. The obtained geometry parameters are given in Table 1, which also lists the values for an optimization using TDDFT. As can be seen, the LVC model does not fully reproduce the excited-state minima, giving a much shorter long S–O bond than TDDFT. We ascribe this deficiency (i) to the fact that the S_0_ harmonic oscillator is too stiff for the excited states, (ii) that our model does not take into account the quadratic JT couplings, and (iii) that the S–O bonds are significantly anharmonic in the excited state. However, the main point we want to show here is that LVC models for degenerate states (i.e., for JT and PJT problems) can correctly describe the symmetry-imposed topology of the excited-state PESs, even if they are automatically parametrized using the numerical differentiation scheme, given that an adequate phase correction algorithm is used.

**Table 1:** Optimized Geometry Parameters of SO_3_ in the S_4_ State^a^

value	“SO_3_ LVC”	TDDFT
long S–O distance (Å)	1.597	1.982
short S–O distances (Å)	1.542	1.493
wide O–S–O angle (deg)	126.8	121.2
acute O–S–O angle (deg)	116.6	119.4

a Both optimized geometries are planar (*C*_2*v*_ symmetry).

### LVC Parameter Fidelity for [PtBr_6_]^2–^

In this section, we compare the “PtBr good” and “PtBr poor” models that were described above. These models only differ in (i) the magnitude of the displacements used during numerical differentiation (0.10–0.15 for “PtBr good” and 0.05 for “PtBr poor”) and (ii) the wave function truncation threshold (0.999999 for “PtBr good” and 0.998 for “PtBr poor”).

The individual κ and λ parameters for the two models are compared in detail in Section S1 of the Supporting Information. Figure S1 evidences that the “PtBr poor” model has numerous very large, spurious λ parameters. Many of these parameters do not conform with the symmetry-imposed selection rules, e.g., that a normal mode of *u* symmetry should not couple states of equal parity (*u* – *u* or *g* – *g*). Overall, the amount of symmetry-breaking parameters and very large parameters (magnitude of >0.001 au) raises red flags regarding the reliability of the parameters of the therefore so-called “PtBr poor” model. In contrast, as shown in Figure S1, the “PtBr good” model conforms to symmetry and has much fewer large parameters. Section S1 also contains a further discussion that compares the LVC parameters of the “PtBr good” model with those from ref (25) (in Figure S2), demonstrating that these two models are consistent with each other. Furthermore, Figure S3 provides evidence that, for [PtBr_6_]^2–^, high-fidelity wave function overlaps seem to be somewhat more important than frequency-dependent displacement magnitudes to obtain accurate LVC models, although these magnitudes also affect the quality of the model.

**Figure 1:**
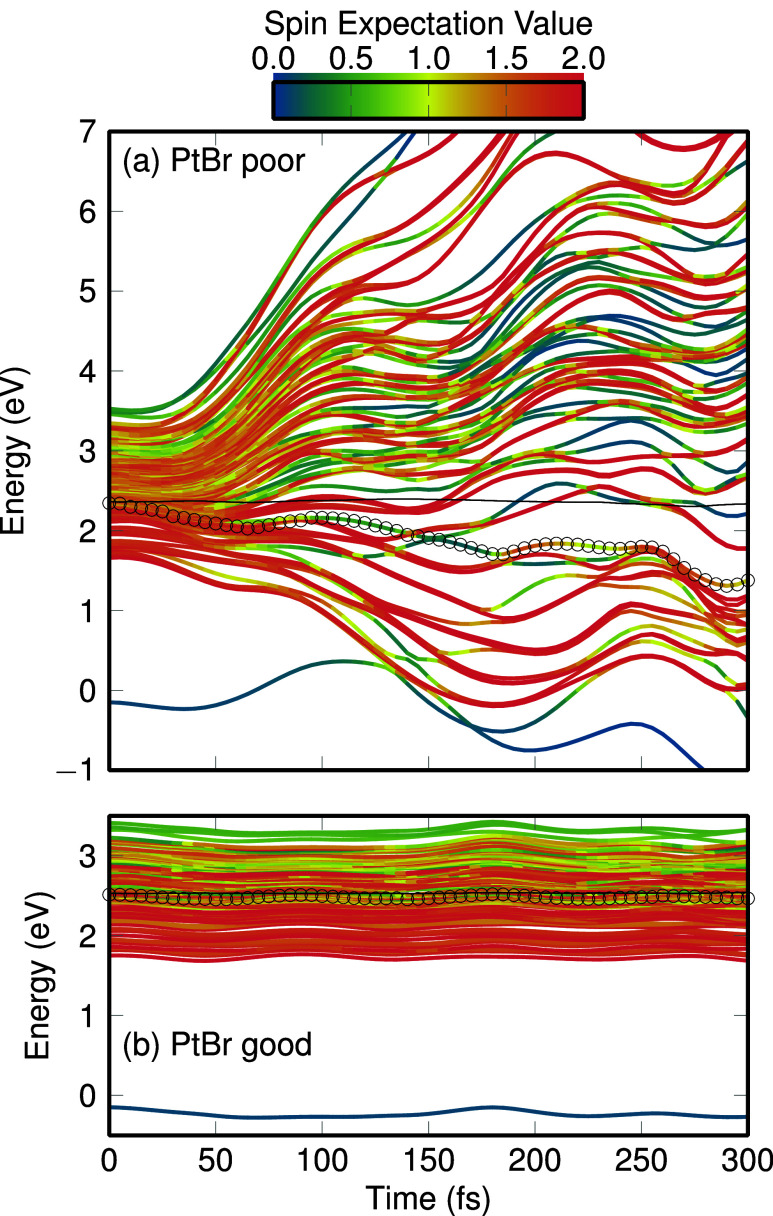
Time evolution of the potential energies along an LVC trajectory of [PtBr_6_]^2–^ simulated with the (a) “PtBr poor” and (b) “PtBr good” models. Each line corresponds to one diagonal^9^ state (i.e., eigenstate of the spin–orbit Hamiltonian). The color denotes the spin expectation value of the state, with 0 corresponding to pure singlet and 2 to pure triplet. The dots indicate the current active state. The thin black line indicates the total energy.

Figure 1 shows an example trajectory of [PtBr_6_]^2–^ simulated with the “PtBr poor” and “PtBr good” models, clearly illustrating how spurious LVC parameters can affect the dynamics. Panel (a) displays the trajectory that uses the “PtBr poor” model. In the first 20 fs, all 96 excited states (linear combinations of the 24 singlets and 3 × 24 triplet components) form a band of roughly 1.5 eV width, where the strong spin–orbit coupling is evident from the extensive mixing of the states, indicated by green, yellow, and orange colors. Problematically, starting around 30–40 fs, the PESs start to quickly diverge, with the lower states decreasing in energy and the higher states increasing. This behavior persists until the end of the 300 fs simulation time, at which point the states span a bandwidth of more than 11 eV.

Since all excited states have qualitatively the same electronic structure (ligand-to-metal charge transfer from Br *p* orbitals to Pt *e*_*g*_ orbitals), it is unexpected for the states to spread apart to such an extent. The reason for the diverging states are the large λ parameters in the “PtBr poor” model (cf Section S1). Once the atoms move away from the reference geometry *Q⃗* = 0, the spuriously large λ parameters produce large electronic couplings λQ between the various states, leading to diverging eigenstates. As the active state is one of the lower-lying states that gets stabilized by these spurious interactions, the atoms move very far away from the initial geometry, increasing the couplings even further in a positive feedback loop. Overall, a physically unreasonable dynamics with excessive molecular distortion ensues due to the large λ parameters.

Figure 1b shows a trajectory based on the same initial conditions as in panel a, but using the “PtBr good” model, and identical settings otherwise. Here, across the entire 300 fs simulation time, all 96 excited states remain as a narrow band of states, without any diverging behavior. Nuclear motion is significantly reduced, as only the totally symmetric (*a*_1*g*_) breathing mode is notably activated. This is in contrast to the “PtBr poor” model, where essentially all modes exhibit large-amplitude motion. Further details and comparisons to full quantum dynamics simulations for [PtBr_6_]^2–^ can be found in ref (25).

### LVC Excited-State Dynamics of [Ru(bpy)_3_]^2+^

This subsection presents the results for coupled electron–nuclear dynamics of [Ru(bpy)_3_]^2+^, starting with the absorption spectrum that defines the initial conditions, followed by the evolution of the electronic populations and of the most relevant nuclear degrees of freedom. In all subsections, we put the main focus on comparing the LVC and TDDFT results.

#### Absorption Spectrum

**Figure 2:**
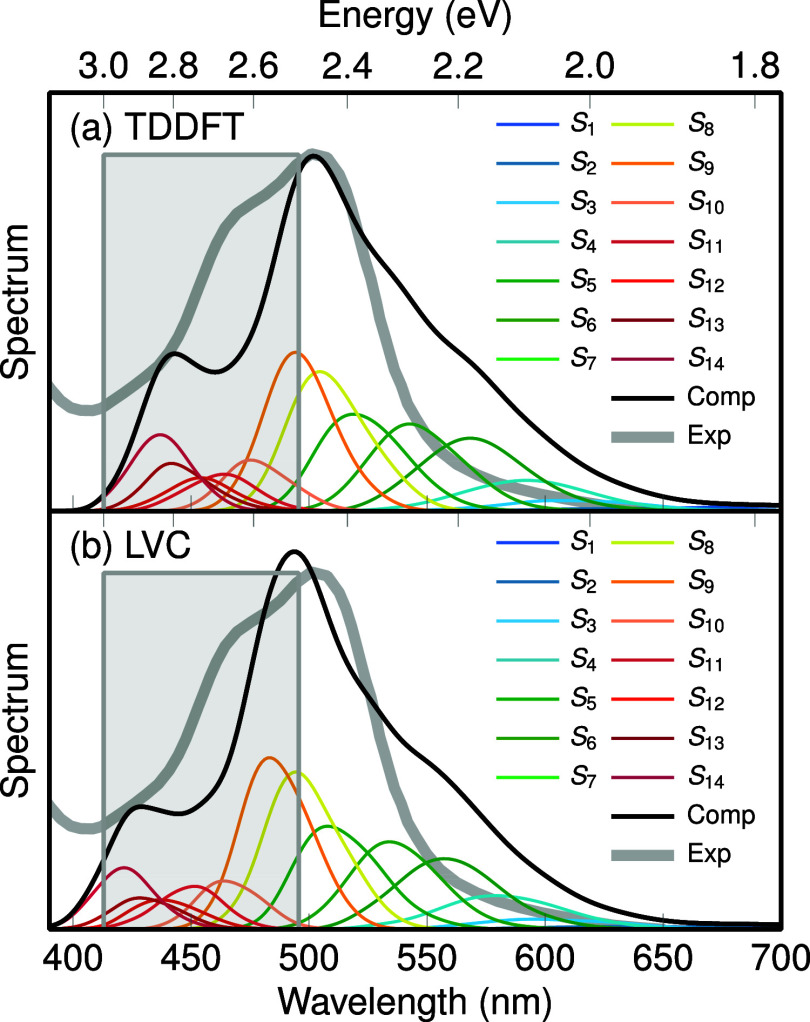
UV–vis (a) TD-DFT and (b) LVC absorption spectrum of [Ru(bpy)_3_]^2+^ calculated from a Wigner distribution of 500 geometries and Gaussian convolution (full-width-at-half-maximum of 0.1 eV, black line).^46^ Colored lines indicate the contributions of each adiabatic state (S_1_–S_14_). The gray line indicates the experimental absorption spectrum in aqueous solution,^55^ shifted by −0.27 eV. The experimental spectrum is reproduced from ref (55). (Copyright 2015, American Chemical Society). The light gray box denotes the excitation window for the nonadiabatic dynamics simulations.

Figure 2 shows the calculated absorption spectra of [Ru(bpy)_3_]^2+^, generated from a Wigner distribution including 500 initial geometries and excitation energies for 14 electronic excited singlet states, using directly TDDFT (a) or LVC (b). Reassuringly, both spectra are very similar and exhibit a very weak band between 750 and 650 nm, a main band raising from about 650 nm, a weak shoulder around 560 nm, and a band maximum at 500 nm. There is also a small peak at about 460 nm, although we expect that it would change into the onset of the second absorption band if more states were calculated. The agreement between the TDDFT and LVC spectra is very good, with a small 10 nm red-shift of the TDDFT spectrum compared to the LVC one. As TDDFT and LVC produce identical energies and oscillator strengths for *Q⃗* = 0 by construction, this shift can be assigned to state-specific frequencies and normal modes (i.e., quadratic vibronic coupling terms) and anharmonicities, both of which are not present in the LVC model. The obtained spectra also agree very well with the one previously computed by us,^39^ which used a very similar level of theory. Furthermore, a decent agreement with the experimental absorption spectrum in aqueous solution^55^ is found, although the experimental absorption is slightly (+0.27 eV) blue-shifted relative to computed spectra.

Based on our spectra, the first absorption band spans from the S_1_ to the S_12_, and the onset of the second band starts at the S_13_. All of these states have metal-to-ligand charge transfer character, dominated by excitations from the three occupied 4*d* orbitals to linear combinations of the two lowest π* orbitals on the three bpy ligands.^56^ Using an excitation window of 2.5–3.0 eV (gray box in Figure 2, chosen to match ref (27)), we primarily excite S_8_ to S_14_.

#### Electronic Populations

**Figure 3:**
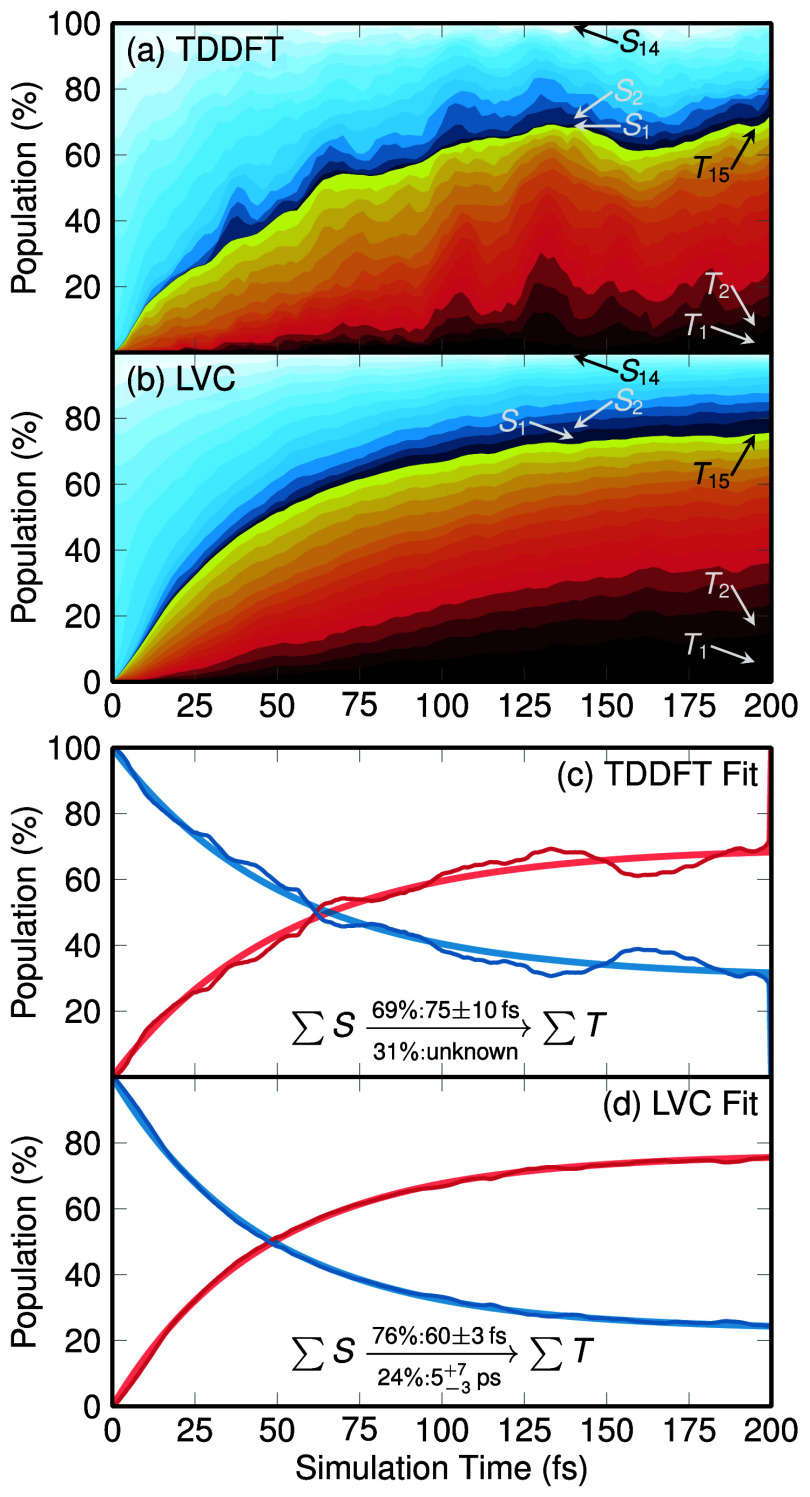
Evolution of electronic (adiabatic) populations. Panels (a) and (b) show the population of all included states (*S*_1_ to *S*_14_ in blue/white, *T*_1_ to *T*_15_ in red/yellow) for the TDDFT and LVC trajectories, respectively, as stacked area plots. Panels (c) and (d) show the total singlet and triplet populations and biexponential kinetic model fits. Note that for (c) a second time constant could not be estimated from the short (200 fs) TDDFT trajectories, whereas for (d) the longer (1000 fs) LVC trajectories permitted a proper biexponential fit. The electronic populations and biexponential fit are plotted to 1000 fs in Figure S3.

Figure 3 presents the time evolution of the electronic populations according to the TDDFT and LVC simulations. The TDDFT results are based on 53 trajectories, the LVC ones on 582 trajectories. These results are shown until 200 fs, although the LVC simulations were run for 1 ps (See Section S2). Panels a and b show the time evolution of the spin-free electronic populations. Due to the large number of states, we show the populations as stacked area plots, where the vertical width of each color indicates the population of the corresponding state. The initially excited population is distributed over the S_7_—S_14_ states. Rather than decaying to the low-lying singlet states, due to the large spin–orbit couplings, the singlet population undergoes efficient (“horizontal”) intersystem crossing (ISC), reaching 50% triplet population at 60 fs (TDDFT) or 50 fs (LVC) and continuing to grow after that time. Simultaneously, the population is also somewhat slowly nonadiabatically relaxing from higher to lower states. After 200 fs, about 80% of the population is in the triplet state, but only half of that is in T_1_ to *T*_4_. It is good to see that the two methods agree qualitatively with each other, although the TDDFT populations rise slightly slower and exhibit some oscillations that are not present in the LVC data. We ascribe the oscillations to larger statistical fluctuations due to the smaller number of trajectories.

Figure 3 also shows the total singlet and triplet populations, together with biexpontial fits. For TDDFT, we obtain a 75 ± 10 fs ISC time constant, which corresponds to 69% of the population. The remaining 31% decays slower, but a statistically significant second time constant could not be obtained due to the limited number of trajectories. For LVC, the fast time constant is 60 ± 3 fs (for 76% of the population), and the slow time constant is approximately 5 ps. Note that the given uncertainties only consider the limited statistics, but not the method-intrinsic errors. We expect that the slow time constant is sensitive to the environment of the complex as well as the initial excitation energy, so that this time constant is not easily comparable to experimental results.

**Figure 4:**
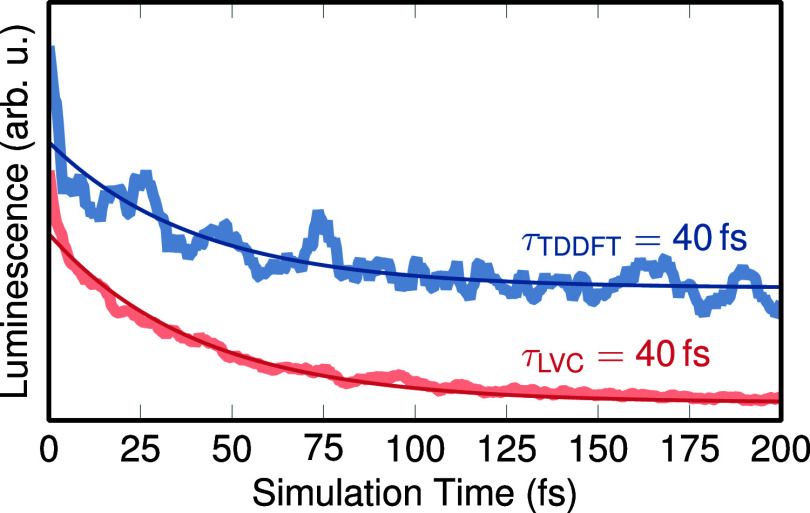
Simulated luminescence decay, computed from the energy differences and oscillator strengths between active state and ground state. Curves were fitted monoexponentially. The TDDFT curve is shifted vertically to show both curves without occlusion.

The predicted fast ISC time constants of 75 or 60 fs are somewhat slower than the experimentally obtained time constants, which include e.g., 40 fs,^57^ 15 ± 10 fs,^58^ 30–45 fs,^59^ or 25 ± 15 fs.^56^ However, most ultrafast spectroscopic experiments are not directly probing the electronic wave function’s spin, but rather rely on the fact that experimental observables (e.g., transient absorption, luminescence decay) are indirect reporters of the electronic spin. As demonstrated multiple times in the literature, see e.g., refs (60−64), the time constants directly extracted from electronic populations of nonadiabatic simulations (such as SH) cannot be compared one-to-one to the time constants observed in experiments, as those include contributions from a probe process. Accordingly, we have simulated the time-dependent luminescence intensity of [Ru(bpy)_3_]^2+^ from the TDDFT and LVC trajectories, presented in Figure 4, and found a time constant of 40 fs for both methods. It is gratifying that this time constant is in agreement with the majority of the experiments,^56,57,59^ so that remaining deviations to other experiments can be attributed to differences in excitation wavelength and/or solvent effects.

#### Nuclear Motion

**Figure 5:**
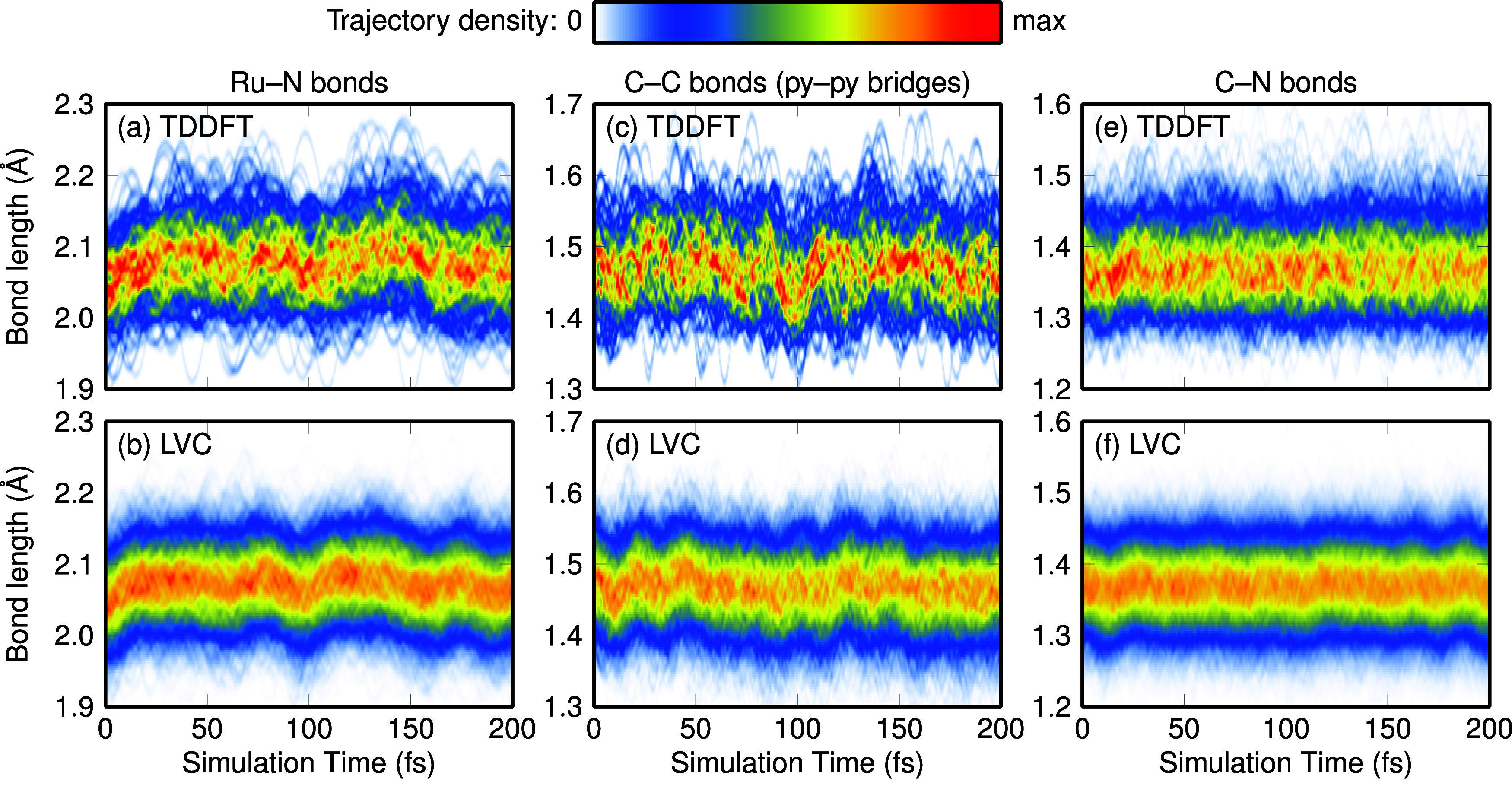
Temporal evolution of (a–b) Ru–N, (c–d) C–C, and (e–f) C–N bond lengths of [Ru(bpy)_3_]^2+^ after excitation. Plots were created from all 53 TDDFT (panels a, c, e) and 582 LVC (panels b, d, f) trajectories by convolution with Gaussians (full-width-at-half-maximum of 0.01 Å). All equivalent bonds (six for Ru–N, three for C–C bridges, 12 for C–N) were included.

Figure 5 presents the time-dependent evolution of the Ru–N, bridge C–C, and C–N bond lengths, as obtained from the TDDFT (panels a,c,e) and LVC (b,d,e) dynamics. The color bar indicates the trajectory density, obtained from Gaussian convolution. Panels a and b compare the Ru–N distances, which are initially distributed around 2.04 Å, and stretch to 2.07–2.08 Å within about 25 fs. After that, they remain approximately constant. The pyridine–pyridine C–C bridges (panels c and d) start at 1.48 Å, then experience weak oscillations (contracting first, then expanding), before the bonds stabilize around 1.47 Å. Finally, the C–N bond lengths (panels e and f) start at 1.37 Å and very quickly move to 1.36 Å. The TDDFT and LVC simulations agree very nicely regarding the presented bond lengths, although the TDDFT results are subject to more noise due to the smaller number of trajectories.

The change of bond lengths in [Ru(bpy)_3_]^2+^ after excitation has been the subject of different investigations, both experimental (see e.g., refs (65,66)) and theoretical (e.g., ref (67)). Experimentally, it has been reported^65^ that the ground state Ru–N distance of 2.066 Å contracted by 0.036 Å (to 2.03 Å) within 50 ps after excitation at 400 nm (3.1 eV). We are not aware of any experimental study that addresses bond-length changes on a fs time scale. Our simulations indicate that the initial, few-fs response of the Ru–N bonds to excitation into the high-energy side of the first absorption band is a slight elongation, with the average bond lengths increasing by about 0.03 Å.

Regarding the long-standing discussion about the localization or delocalization of the excited electron on one/several bpy ligands,^56^ our gas-phase surface-hopping simulations support a delocalized excitation. Neither the TDDFT nor the LVC simulations exhibit any bifurcation in the Ru–N, C–C, or C–N bond lengths that would indicate otherwise. This is in agreement with a detailed meta-analysis from 2017^56^ and also from a recent computational work that reported barrierless electron hopping.^67^

Overall, the agreement of the LVC and TDDFT trajectories—in terms of absorption spectrum, electronic populations, and nuclear motion—is very good. This clearly indicates that our LVC model for [Ru(bpy)_3_]^2+^ was adequately parametrized in the presence of degenerate *E* states due to its *D*_3_ symmetry and it can properly describe its dynamics. Additionally, the results show that [Ru(bpy)_3_]^2+^ is a very rigid system and therefore the employed LVC models are capable of describing its dynamics well, even over the course of hundreds of femtoseconds. Similar findings were reported before for other transition metal complex systems.^19,68^ We note that the applicability of VC models should still in each case be scrutinized carefully.

### General Considerations for LVC Parametrization

At this point, it is important to emphasize that obtaining accurate LVC parameters using the numerical differentiation approach in SHARC requires a careful control of the precision of the involved electronic structure calculations and the step size for the displacements. This is because the λ parameters are computed by dividing small energy differences by small displacements (optionally, the κ parameters can be computed in the same way). We generally recommend the following, in line with the computational details given above and based on some additional preliminary calculations. The frequency calculation to define *V*_0_ should use large integration grids and very tight thresholds for SCF and optimization. Ideally, explicit symmetry should be used for this frequency calculation. For the excited-state calculations, large integration grids, very tight SCF and Davidson thresholds, large auxiliary basis sets, or whatever is appropriate for the used electronic structure method, should be used.

Furthermore, we recommend that the wave function truncation threshold^69^ is set very close to unity. This threshold controls the number of Slater determinants included in the overlap calculation in order to reduce the computational cost. In the truncation scheme of ref (69), each configuration interaction (CI) vector is sorted by the absolute magnitude of the CI coefficients. Starting with the largest coefficient, all Slater determinants are included in the overlap calculation until the norm of the included determinants exceeds the wave function truncation threshold. Based on the thoughts in ref (70), we expect that errors in λ parameters are roughly proportional to , with *T* being the truncation threshold. If computationally feasible, we recommend using a *T* of at least 0.9997 for LVC parametrizations. Ideally, one should perform some preliminary calculations to estimate the largest *T* that is affordable for their system, given that the computational cost of overlap calculations increases sharply as *T* approaches 1.^70^

**Figure 6:**
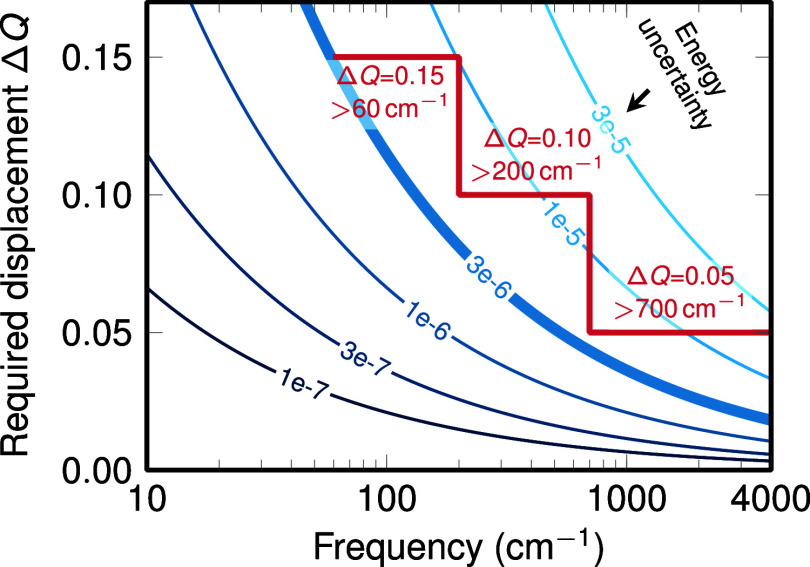
Relationship of normal-mode frequency, uncertainty in the energies computed during LVC parametrization, and the required displacement Δ*Q* to obtain accurate κ and λ parameters. The blue lines plot the relationship Δ*E* = 1/2ω (Δ*Q*)^2^ for different values of Δ*E*. The uncertainty in the electronic energies computed at the displaced geometries is typically on the order of the energy convergence criterion, e.g., of 1 × 10^–6^*E*_*h*_. To obtain adequate κ and λ parameters, here we assume that we should choose the displacement Δ*Q* at least as large as to obtain energy differences of 3 × 10^–6^*E*_*h*_ (thick blue line). Based on this line, we then arrive at reasonable choices of Δ*Q* for different normal-mode frequencies ω.

A final critical aspect of the LVC parametrization is the choice of the displacement magnitude Δ*Q* for each normal mode. Here, experience with previous LVC projects indicated that especially low-frequency modes are prone to spurious λ parameters. The reason is that for these modes, small displacements Δ*Q* will only change the energies of the states by very small amounts, which leads to numerical noise in the computation of the λ parameters. Hence, we recommend that for low-frequency modes the displacement Δ*Q* should be increased. In Figure 6, we present a simple estimation of the displacement Δ*Q* that is needed to obtain a change in energy that is larger than the uncertainty in the energy, which arises from convergence criteria, integration grids, etc. As can be seen, for lower frequencies, larger displacements are needed. Based on Figure 6, we recommend SHARC’s default value of Δ*Q* of 0.05 (in mass- and frequency-weighted normal mode coordinates) for modes above approximately 700 cm^–1^, of 0.10 for modes above approximately 200 cm^–1^, and of 0.15 for lower modes.

### Computational Efficiency

Finally, we would like to highlight the efficiency of LVC simulations. As part of the present work, we improved the efficiency of LVC dynamics simulations through several detailed code optimizations, compared to previous code.^10,16,71^ Within the SHARC–LVC interface, the calculation of gradients and nonadiabatic coupling vectors was sped up through optimizing the contraction order of the involved tensors,^72^ benefiting both LVC and LVC/MM^71^ simulations. Also in the interface, we implemented analytical derivatives of the Kabsch alignment procedure^73,74^ and incorporated the precompilation of critical functions via Numba.^75^ Furthermore, the communication between the pySHARC dynamics driver and the quantum chemistry or LVC interfaces was refactored, now employing the NumPy C-API rather than native Python objects.

These modifications lead to a highly efficient code. In the heaviest and most expensive molecule, [Ru(bpy)_3_]^2+^, each LVC simulation time step (computing energies, spin–orbit couplings, gradients, wave function overlaps, and dipole moments for 16 singlets and 15 triplets) required about 0.118 s on a single core of an Intel Xeon E5–2650 v3 CPU. The total cost of the 582 trajectories, each 1 ps long, amounted to about 38 CPUh (0.065 CPUh/ps). The parametrization of the LVC model involved one optimization plus frequency calculation plus 355 single-point calculations, costing about 2250 CPUh in total. For comparison, the on-the-fly TDDFT simulations required about 1200 s per time step (computing the same quantities) on 16 cores, with an overall cost of 113,000 CPU hours for simulated 10.6 ps (10,660 CPUh/ps). This represents a speedup of more than 5 orders of magnitude of LVC compared to on-the-fly TDDFT, which enables large-scale simulations that would otherwise be unfeasible.

## Conclusions

In this work, we describe how to construct LVC models for rigid molecules possessing degenerate states using a numerical differentiation scheme,^17^ with a focus on the phase correction algorithm. The phase correction algorithm of Zhou et al.^26^ based on “parallel transport” works properly even in cases where states are reordered or heavily mixed, like in states with trigonal or higher symmetry.

We also provide best practices for the underlying electronic structure calculations used in LVC parametrizations in general to ensure accurate *a posteriori* dynamics. Due to the involved numerical differentiation, the quantum chemical calculations needed for the numerical LVC parametrization should be executed with very high precision, including aspects such as integration grids for DFT, convergence criteria for the SCF and Davidson steps, and auxiliary basis sets. Furthermore, the displacement vectors should be sufficiently large, especially for low-frequency modes, where we recommend a value of 0.05 (in mass- and frequency-weighted normal mode coordinates) for modes above 700 cm^–1^, of 0.10 for modes above 200 cm^–1^, and of 0.15 for lower modes. Finally, wave function overlaps should be computed from nearly untruncated CI vectors, even if such wave function calculations might be relatively expensive.^69,70^

These considerations are exemplified in the parametrization of three symmetric systems of increasing size and complexity: SO_3_, [PtBr_6_]^2–^, and [Ru(bpy)_3_]^2+^. In SO_3_, we demonstrate that numerical differentiation can produce LVC parameters that can reproduce the trigonal symmetry of the PESs. The focus in [PtBr_6_]^2–^ is to illustrate how spurious parameters manifest in erroneous SH simulations. Finally, we also present a concise study of the nonadiabatic dynamics of [Ru(bpy)_3_]^2+^ with LVC-parametrized potentials and compare with simulations from direct on-the-fly TDDFT potentials. In agreement with experimental studies, we find evidence that ISC occurs with a sub-100 fs time constant, but this time constant is not identical to the luminescence decay time constant. From our nuclear motion analysis, we find that the Ru–N bonds slightly elongate as a first response to excitation, and that no charge localization occurs within few hundreds of femtoseconds. The LVC simulations on [Ru(bpy)_3_]^2+^ agree very well to the TDDFT reference trajectories, at 5 orders of magnitude lower costs.

In conclusion, we demonstrate that, when properly implemented, within the limits of harmonic potentials, nonadiabatic dynamics on LVC potentials offer a powerful approach to reducing the computational cost associated with the electronic structure of large molecules, such as transition metal complexes, by several orders of magnitude, even in the presence of complicated degenerate state situations. Thus, leveraging LVC potentials enables dynamics in full dimensionality, allowing for an increased number of trajectories, more electronic excited states, and/or the simulation of longer time scales compared to traditional on-the-fly simulations. Future work, expanding LVC models to incorporate anharmonic effects and more complex coupling schemes could further enhance their accuracy and applicability to an even broader range of molecular systems.

## Supporting Information Available

Comparison of LVC parameters for [PtBr_6_]^2–^ with previous work; electronic populations for [Ru(bpy)_3_]^2+^ up to 1 ps from LVC simulations (PDF)
LVC template files for investigated systems (ZIP)
