# Supplementary material for: Parametrization of Linear Vibronic Coupling Models for Degenerate Electronic States
Source: J Phys Chem A. 2025 Mar 4;129(10):2655–66. doi: 10.1021/acs.jpca.4c07472 (PMC11912485; doi:10.1021/acs.jpca.4c07472)
Supplement: Supplementary file 1 — jp4c07472_si_001.pdf [file jp4c07472_si_001.pdf]

Supporting Information: On the Parametrization of Linear Vibronic Coupling Models for Degenerate Electronic States

Dilara Farkhutdinova,[†,‡] Severin Polonius,[†,‡] Paul Karrer,[†] Sebastian Mai,[*,†] and Leticia González[*,†,¶]

†*Institute of Theoretical Chemistry, Faculty of Chemistry, University of Vienna, Währinger Straße 17, 1090 Vienna, Austria*

‡*Vienna Doctoral School in Chemistry (DoSChem), University of Vienna, Währinger Straße 42, 1090 Vienna, Austria*

¶*Research Platform on Accelerating Photoreaction Discovery (ViRAPID), University of Vienna, Währinger Strasse 17, 1090 Vienna, Austria*

E-mail: sebastian.mai@univie.ac.at; leticia.gonzalez@univie.ac.at

Contents

S1 Comparison of LVC model parameters for $[PtBr_6]^{2-}$ **S-2**

S2 Electronic populations of $[Ru(bpy)_3]^{2+}$ up to 1 ps **S-7**

References **S-7**

S1 Comparison of LVC model parameters for $[PtBr_6]^{2-}$

In Figure S1, we compare the LVC parameters between the "PtBr good" and "PtBr poor" models. The only differences between these two models are (i) that "PtBr good" used displacements of 0.10–0.15 (depending on frequency) while "PtBr poor" uses 0.05, and (ii) that "PtBr good" uses a wave function truncation threshold of 0.999999 and "PtBr poor" of 0.998. As evidenced by the figure, the poor model shows severe symmetry violations, which we will discuss normal mode by normal mode.

The t_{2u} and t_{1u} modes (1–3, 7–9, 13–15; left, middle, and right column) are expected to have κ values of zero, λ values of zero between states of the same parity ($g - g$ or $u - u$), and rather small λ values between states of different parity ($g - u$). The "PtBr good" model fully meets these expectations, showing only a block of non-zero parameters between the lower-energy g states and the higher-energy u states. The "PtBr poor" model also correctly exhibits zero κ parameters, as these do not depend on the displacements or wave function truncation threshold. However, the "PtBr poor" model shows extremely large λ parameters, with magnitudes of up to 0.01 (the largest λs for u modes in the "PtBr good" model are one order of magnitude smaller).

The t_{2g} modes (4–6; second column) are analogously expected to have κ values of zero, λ values of zero between states of different parity ($g - u$), and rather small λs between states of the same parity ($g - g$ or $u - u$). Both models produce several non-zero, but still small, κ parameters, which seem to arise from some small symmetry-breaking components in the TDDFT analytical gradients. The λ parameters between states of different parity are correctly zero for both models, but the same-parity λ parameters are one or even two orders of magnitude too large in the "PtBr poor" model, especially in mode 4.

The e_g modes (10–11; fourth row) are expected to have non-zero κ values, λ values of zero between states of different parity ($g - u$), and large λs between states of the same parity ($g - g$ or $u - u$). The large, non-zero κ and λ values are primarily responsible for the JT and PJT effects in the complex and thus significantly shape the PES landscape. Interestingly, the e_g modes are the only modes where the "PtBr good" and "PtBr poor" models agree qualitatively and fully conform to the symmetry of the complex.

The totally symmetric a_{1g} (12; also fourth column) is expected to have large, non-zero κ values for all states, but zero λ values. We find a good agreement between the models for the κ parameters. The "PtBr good" model exhibits only very few very small λ parameters (as expected), whereas the "PtBr poor" model exhibits a large number of λ values that are up to 0.002 in magnitude.

Overall, there are major differences between the two models, with the "PtBr poor" model not only breaking the expected symmetry, but also exhibiting a large number of very large λ parameters, which lead to the instability in the trajectory presented in the main text.

In Figure S2, we compare the "PtBr good" model with the one from Ref. S1. Both models conform to the symmetry, with the model from Ref. S1 showing a smaller overall number of non-zero parameters. Considering that the magnitudes of individual parameters cannot be compared due to different, arbitrary mixing among the degenerate modes and the degenerate states, the two models are consistent and seem to not contain spurious couplings that lead to diverging potential energy surfaces.

In Figure S3, we furthermore show the parameters of two models that are intermediate between the "PtBr good" and "PtBr poor" models. One of the models (top left triangles) in Figure S3 uses the high fidelity overlaps from "PtBr good" with the uniform displacements of "PtBr poor", while the other model (bottom right triangles) uses the poor overlaps from "PtBr poor" but the improved displacements from "PtBr good". As can be seen, the model with the high-quality overlaps shows good conformity with symmetry and few large parameters, even though only using default displacements. In contrast, the model with mixed displacements and poor overlaps shows some weak symmetry violations and some large coupling parameters. However, the latter model still seems to be more reasonable than "PtBr poor" shown in Figure S1. Hence, we recommend that both frequency-dependent displacements as well as high-quality overlaps should be employed when parametrizing LVC models.

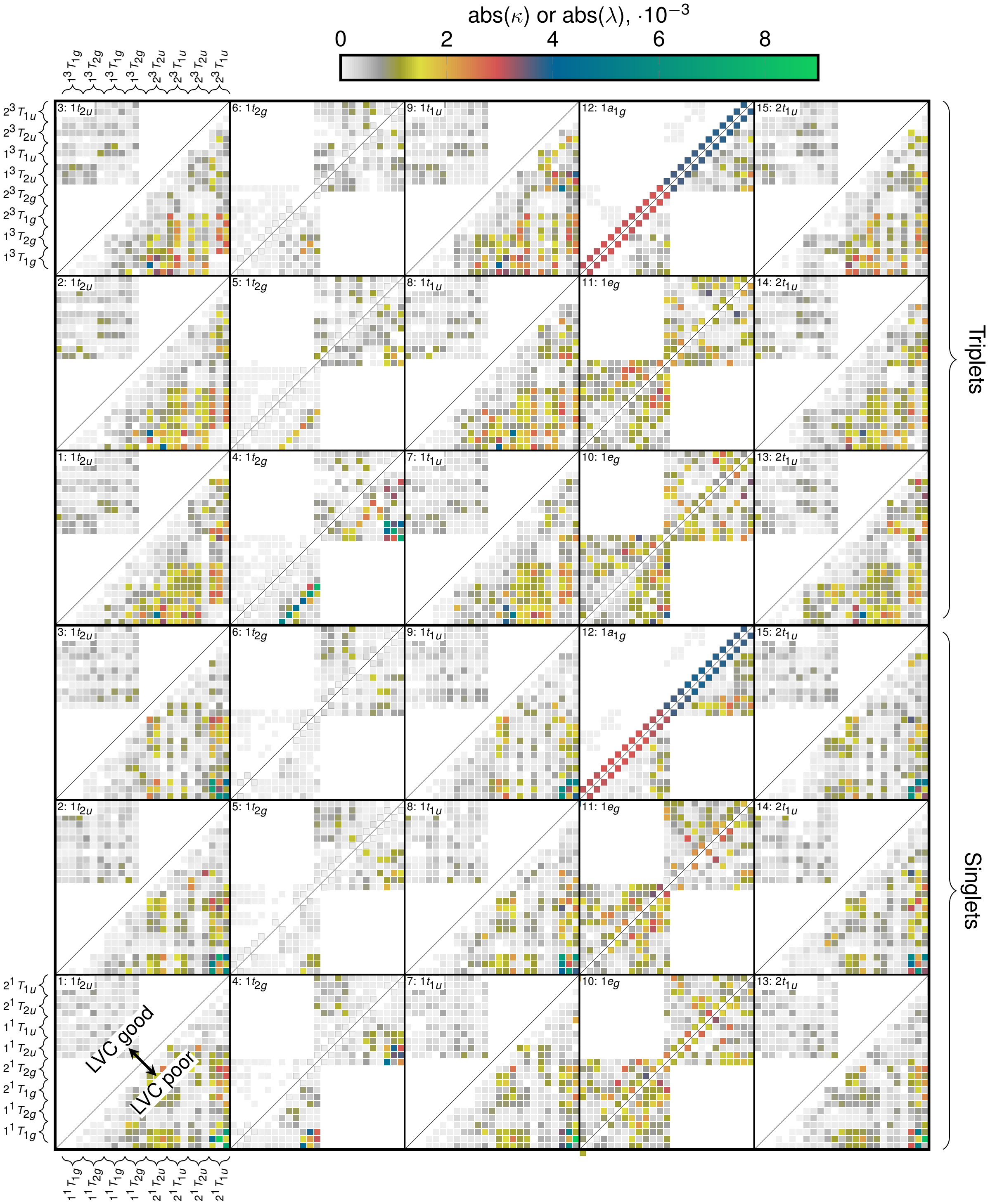

Figure S1: Comparison of the LVC parameters $\kappa_i^{(\alpha)}$ and $\lambda_i^{(\alpha\beta)}$ for $[PtBr_6]^{2-}$ of the "PtBr good" and "PtBr poor" models. The lower half compares the parameters for 24 singlet states, the upper half for 24 triplet states (see bottom/top left for the state labels). Each square compares all parameters for one normal mode i (labels are in each top left corner). Within each square, data above the diagonal corresponds to the current model, below the diagonal to Ref. S1. The absolute magnitude of each parameter is given by a colored square (see color bar); parameters $< 10^{-5}$ a.u. are not drawn. The $\kappa_i^{(\alpha)}$ parameters are drawn directly next to the diagonals, and the $\lambda_i^{(\alpha\beta)}$ further away from the diagonals.

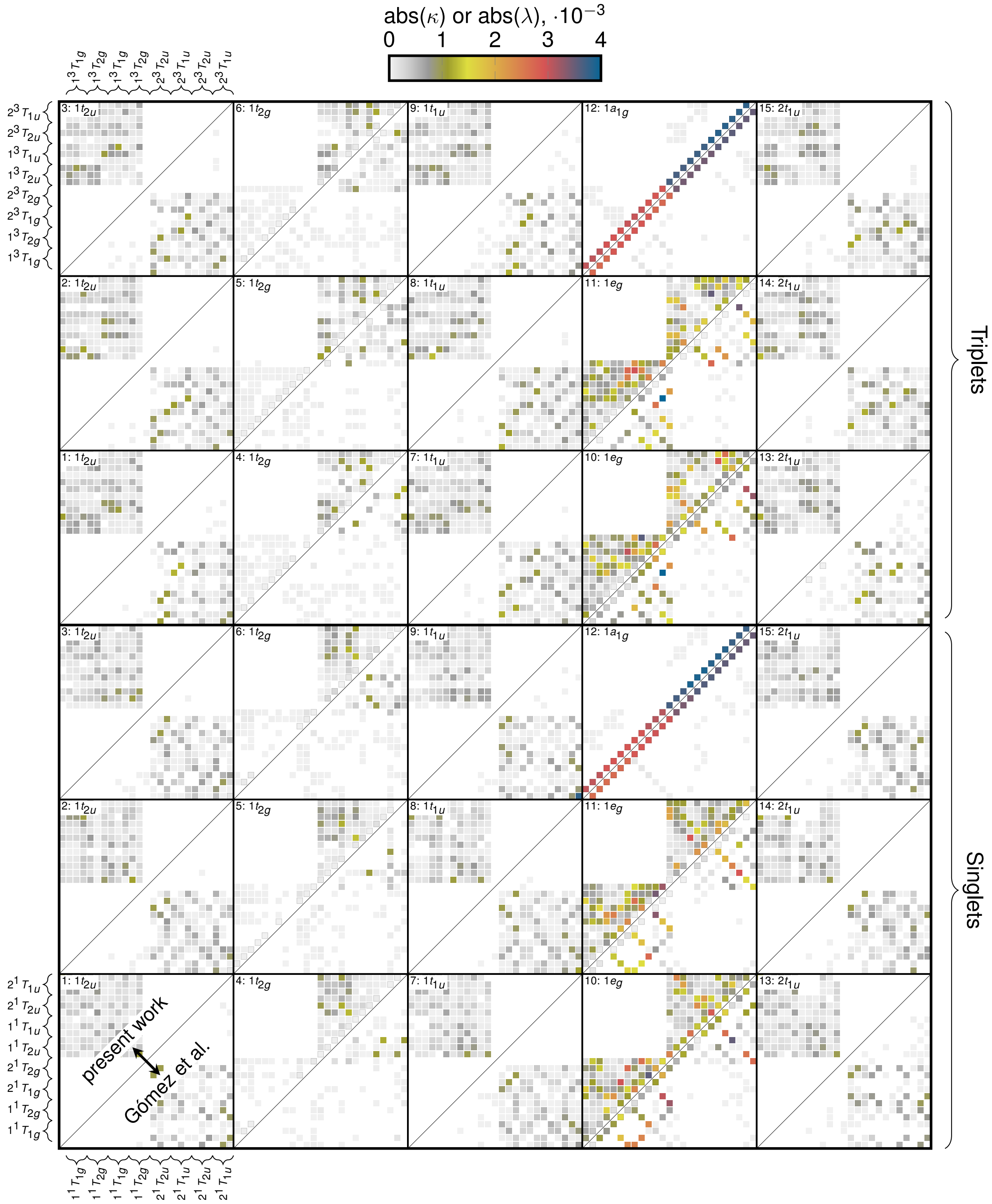

Figure S2: In the same style as in Figure S1, a comparison of parameters between the "PtBr good" model and the model from Ref. S1.

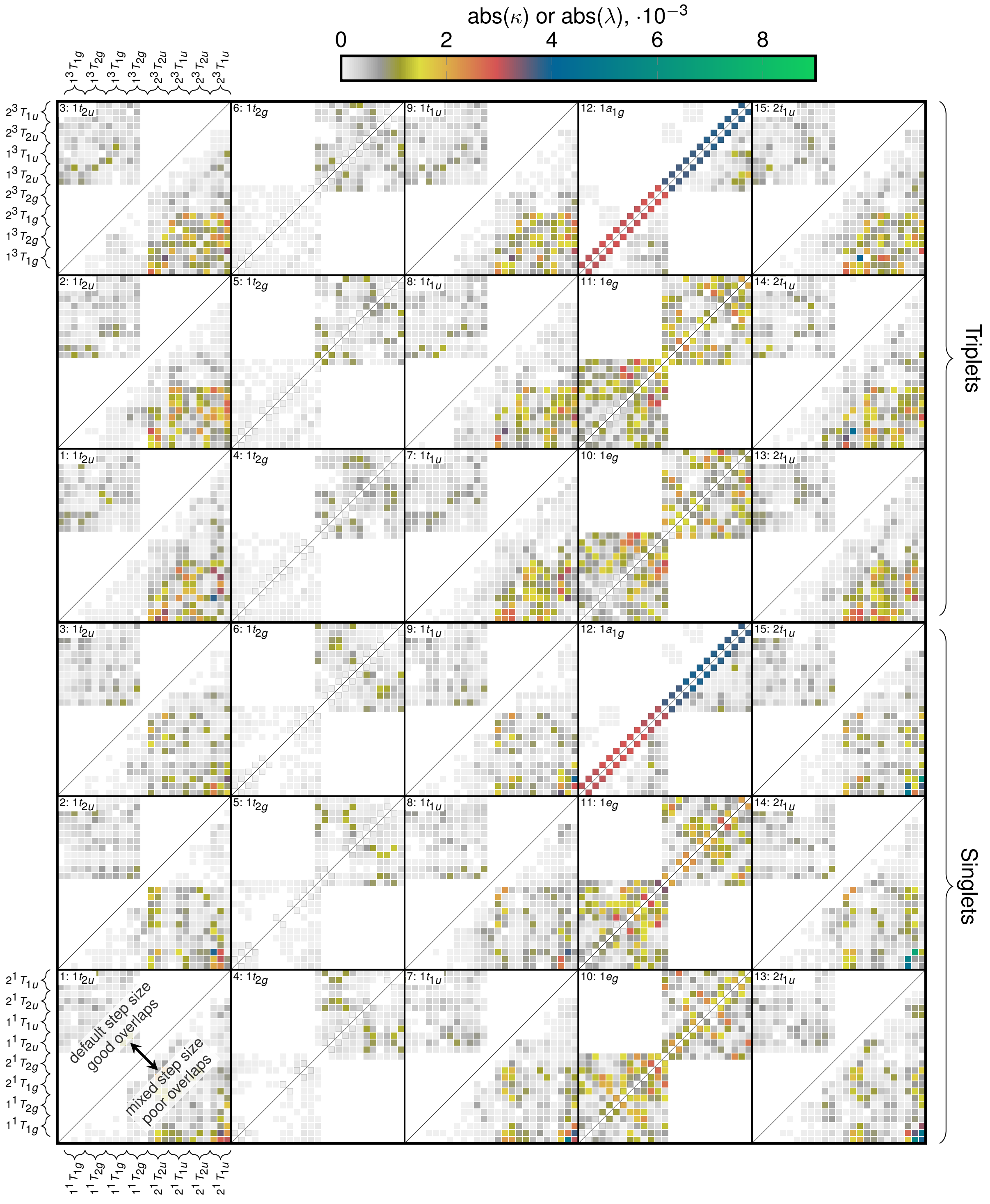

Figure S3: In the same style as in Figure S1, a comparison of parameters of two additional models derived from the "PtBr good" model, but the top left parameters use uniform displacements of 0.05 (instead of mixed displacements depending on frequency) and the bottom right parameters use an overlap threshold of 0.998 (instead of 0.999999).

S2 Electronic populations of $[Ru(bpy)_3]^{2+}$ up to 1 ps

In Figure S4, we display the electronic populations from the SHARC trajectories propagated with the "Rubpy LVC" model up to 1 ps. Note the slowly increasing triplet yield after 200 fs.

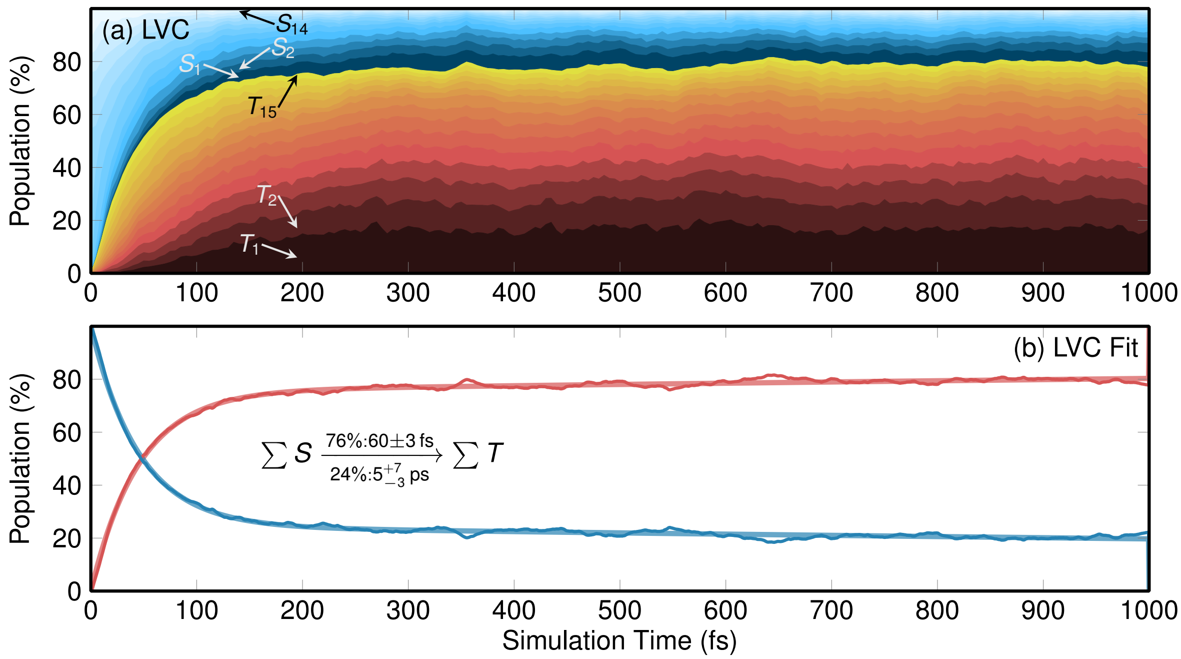

Figure S4: Evolution of electronic (adiabatic) populations of the 582 trajectories simulated with the "Rubpy LVC" model. Panel (a) shows the population of all included states (S_1 to S_{14} in blue/white, T_1 to T_{15} in red/yellow). Panel (b) shows the total singlet and triplet populations and a biexponential kinetic model fit. This plot shows the same data as Figure 4b and d in the main manuscript, except that the data is shown to 1000 fs.

References

(S1) Gómez, S.; Heindl, M.; Szabadi, A.; González, L. From Surface Hopping to Quantum Dynamics and Back. Finding Essential Electronic and Nuclear Degrees of Freedom and Optimal Surface Hopping Parameters. *J. Phys. Chem. A* **2019**, *123*, 8321–8332.
